# Comparison of Microbiota Variation in Korean Healthy Adolescents with Adults Suggests Notable Maturity Differences

**DOI:** 10.1089/omi.2018.0146

**Published:** 2018-12-19

**Authors:** Joo-Wook Kim, Jin Sook Lee, Jung Ho Kim, Joo-Won Jeong, Dae Ho Lee, Seungyoon Nam

**Affiliations:** ^1^ College of Medicine, Gachon University, Incheon, Korea.; ^2^ Gachon Institute of Genome Medicine and Science, Gachon University Gil Medical Center, Incheon, Korea.; ^3^ Department of Pediatrics, Genome Medicine and Science, Gil Medical Center, Gachon University College of Medicine, Incheon, Korea.; ^4^ Department of Internal Medicine, Gachon University Gil Medical Center, Gachon University School of Medicine, Incheon, Korea.; ^5^ Department of Anatomy and Neurobiology, College of Medicine, Kyung Hee University, Seoul, Korea.; ^6^ Department of Biomedical Science, Graduate School, Kyung Hee University, Seoul, Korea.; ^7^ Gachon Advanced Institute of Health Sciences & Technology, Gachon University, Incheon, Korea.; ^8^ Department of Life Sciences, Gachon University, Seongnam, Korea.

**Keywords:** population microbiome science, Korean adolescents, co-occurrence network, Human Microbiome Project, developmental biology

## Abstract

Comparative studies of microbiome variation in world populations and different developmental stages of organisms are essential to decipher the linkages among microbiome, health, and disease. Notably, the gut microbiota are believed to mature in early life. In this context, we compared the gut microbiota diversity in Korean adolescent healthy samples (KAHSs) to healthy Korean adults (HKAs) as well as the Human Microbiome Project healthy samples (HMPHSs), the latter being one of the largest adult cohorts, based on organismal composition, alpha- and beta-diversities, function/pathway prediction analysis, and co-occurrence networks. We found that the gut microbiota compositions, including the ratios of firmicutes to bacteroidetes, between KAHSs and HMPHSs were different, and the diversities of KAHSs were less than those of HMPHSs. The predicted functions, for example, secondary bile acid synthesis and insulin signaling of KAHSs and HMPHSs, were also significantly different. Genus-level networks showed that co-occurrences among different taxa more frequently happened in HMPHSs than in KAHSs. Even though both KAHSs and HMPHSs represent healthy microbiomes, comparisons showed substantial differences, likely implicating different diets, environments, and demographics. Interestingly, we observed lower microbial diversities and less frequent co-occurrences among different taxa in KAHSs than adult HMPHSs and HKAs. These new findings collectively suggest that the adolescent gut microbiota in the present Korean sample did not reach the extent of maturity of adult microbiota diversity. In all, further population studies of microbiome variation across geographies and developmental stages are warranted, and should usefully inform future diagnostics and therapeutics innovation targeting the microbiome.

## Introduction

The human gut microbiota facilitates numerous vital biological processes, including energy harvesting, metabolism of short-chain fatty acids (SCFAs), and inflammation, and its dyshomeostasis contributes to diverse diseases such as obesity, neurodegeneration, and cancer (Bonfili et al., 2017; Cani, 2018; Dimitrov, 2011; Houser and Tansey, 2017; Human Microbiome Project Consortium, 2012; Jiao et al., 2018; Kau et al., 2011; Koliada et al., 2017; Malan-Muller et al., 2018; Sonnenburg and Backhed, 2016; Zeller et al., 2014).

Recently, high-throughput sequencing technology has enabled determination of the gut microbiome composition (Laudadio et al., 2018; Nishijima et al., 2016; Tsai et al., 2015), allowing for analysis of individual organismal proportions, as well as microbiota diversity, which associates with good health (Sonnenburg and Backhed, 2016). These techniques have been applied to gut microbiota studies for diverse large cohorts of healthy subjects, and those suffering from rheumatoid arthritis, diabetes, and colorectal cancer, in Europe, United States, Russia, and Asia (Backhed et al., 2015; Forslund et al., 2015; Karlsson et al., 2013; Nakayama et al., 2015; Nishijima et al., 2016; Qin et al., 2010, 2012, 2014; Turnbaugh et al., 2009; Tyakht et al., 2013; Zeller et al., 2014; Zhang et al., 2015).

In Korean cohorts, gut microbiome characterization is now underway, primarily of healthy adults or those with various disease states (Hu et al., 2015; Kim et al., 2013, 2014; Nam et al., 2011; Song et al., 2014; Yun et al., 2017).

Notably, the adolescents experience rapid changes, physically and physiologically, in human development (Christie and Viner, 2005). The changes during adolescence are often affected by individual factors (e.g., sex) and environmental factors (e.g., undernutrition, substance use) (Christie and Viner, 2005). Adolescent development changes the disease aspects in reproductive health problems, injuries, and mental illness (World Health Organization, 2006). Also, health-related behaviors often affect long-term outcomes on present and future health (World Health Organization, 2006). Hence, comparative studies of microbiome variation in world populations and developmental stages of organisms are essential to decipher the linkages among microbiome, health, and disease.

In this study, Korean adolescents (Hu et al., 2015) were compared with adult cohorts, including the Human Microbiome Project (HMP), one of the largest healthy adult samples (Human Microbiome Project Consortium, 2012; Turnbaugh et al., 2007), and Korean adult samples (Yun et al., 2017), in terms of gut microbiota. Through the comparison, we have not only examined the gut microbiota differences, but also observed whether or not gut microbial diversity of the adolescents reached to the extent of diversity of healthy adults. By comparing microbiome composition, microbiota diversities, function/pathway analysis, and co-occurrence networks, we developed a deeper understanding of the Korean healthy adolescents' gut microbiota specifically, and with a view to developmental biology broadly.

## Materials and Methods

### Datasets and preprocessing

The Korean adolescent healthy samples (KAHSs) (Hu et al., 2015), Human Microbiome Project healthy samples (HMPHSs) (Human Microbiome Project Consortium, 2012), and healthy Korean adults (HKAs) (Yun et al., 2017) are described in Table 1. An overview of the data analysis workflow is shown in Supplementary Figure S1. The HMPHSs are available at https://portal.hmpdacc.org, and the KAHSs (Hu et al., 2015) were downloaded from http://metagenome.cafeomics.com/public/download.php

**Table 1.** Description of the Datasets in the Present Study

*Dataset*	*Raw reads (median)*	*Reads after filtering low quality and short reads*	*Cleaned reads*	*Sequencing platform*
KAHSs (*n* = 67)	9958	3761	2861	GS Junior
HMPHSs (*n* = 325) (processed data used)	—	13974	10884	GS FLX
HKAs (*n* = 1463)	42,379	10,940	12,405	Illumina Miseq
IAHSs (*n* = 10)	14,285	5606	5398	GS FLX

HKAs, healthy Korean adults; HMPHSs, Human Microbiome Project healthy samples; IAHSs, Italian adolescent healthy samples; KAHSs, Korean adolescent healthy samples.

Low-quality KAHS reads (average quality score below 30, and <250 bp) were filtered out using fastx_toolkit 0.014 (hannonlab.cshl.edu/fastx_toolkit). Chimera detection and removal were performed with vsearch (v2.4.3) (Rognes et al., 2016) and the Ribosomal Database Project (RDP) training dataset (release 16_022016) (Cole et al., 2014) guided by the Microbiome_helper (Comeau et al., 2017).

The HKAs (Yun et al., 2017) were downloaded from CODA (Clinical & Omics Data Archive) available at coda.nih.go.kr/coda We merged raw reads of HKAs using Paired-End reAd mergeR (PEAR; version 0.9.6) (Zhang et al., 2014) and filtered low-quality reads (less than average quality score 25, and <400 bp) using fastx_toolkit 0.014. The same procedure above was applied to chimera removal for the reads in the HKAs.

Italian adolescent healthy samples (IAHSs) (Del Chierico et al., 2018) were obtained from the NCBI BioProject accession PRJNA280490 (www.ncbi.nlm.nih.gov/bioproject). KAHSs were age 13–16 years (13.8 ± 3), and IAHSs 13–19 years (16.9 ± 1.1).

The data used in the present study were drawn from four public datasets (Table 1) that have been approved by their own institutional Ethics Committees (Del Chierico et al., 2018; Hu et al., 2015; Human Microbiome Project Consortium, 2012; Yun et al., 2017).

### 16S ribosomal RNA sequence analysis

Operational Taxonomic Unit (OTU) clustering was done by SortMeRNA (version 2.1b) (Kopylova et al., 2012), contained in Quantitative Insights into Microbial Ecology (QIIME) (Caporaso et al., 2010) in a closed reference approach (97% identity threshold). OTU sequences were assigned to the Greengenes database (release 13_8) (DeSantis et al., 2006) with UCLUST (version 1.2.22q) (Edgar, 2010), inside QIIME. We subsampled 3000 reads from each sample, to rarify for diversity analysis in QIIME (Caporaso et al., 2010). Subsequently, we estimated alpha-diversity and beta-diversity in QIIME (Caporaso et al., 2010).

### Functional analysis

Functional profiling prediction was performed with Phylogenetic Investigation of Communities by Reconstruction of Unobserved States (PICRUSt; version1.1.3) (Langille et al., 2013), and was visualized by STAMP (version 2.1.3) (Parks et al., 2014). Taxonomy composition plot and rarefaction curves were made using R (version 3.4.3) and QIIME.

### Network analysis

For phylum-level network analysis, SPIEC-EAST (Kurtz et al., 2015) was used, and CoNet (Faust and Raes, 2016) was used to generate genus-level networks for the two populations. Low abundant taxa, with <20 reads across the samples, were removed. The thresholds for Pearson correlation were set to 0.7 for both positive and negative values. Other than those, we went with the default options.

## Results

### Comparisons of KAHSs and HMPHSs stool samples

**FIG. 1.**
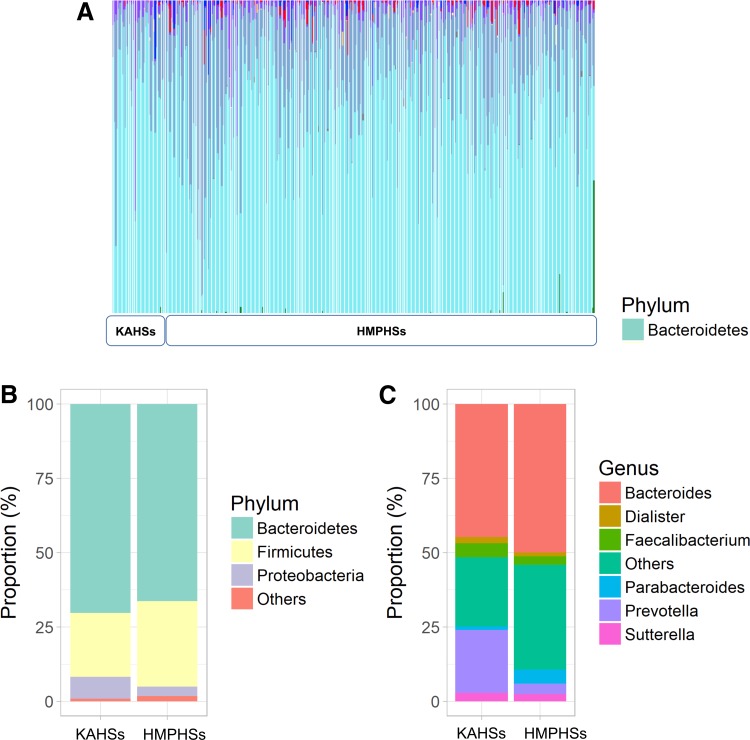
Gut microbiome composition of KAHSs and HMPHSs, at the phylum and genus levels. **(A)** Phylum-level taxonomy proportion plot for all samples, including KAHSs and HMPHSs. **(B)** Taxonomy proportion plots for the phylum level. **(C)** Taxonomy proportion plots for the genus level. HMPHSs, Human Microbiome Project healthy samples; KAHSs, Korean adolescent healthy samples.

To characterize the stool microbiota of KAHSs, we compared samples to those of adult HMPHSs, showing both to possess prevalent cyan-colored species (bacteriodetes) (Fig. 1A). At the phylum level, bacteroidetes amounted to 70.3% and 66.4% of KAHSs and HMPHSs, respectively (Fig. 1B), whereas firmicutes amounted to 21.5% and 28.7% in KAHSs and HMPHSs, respectively. At the genus level of KAHSs and HMPHSs, respectively, bacteroidetes amounted to 44.7% and 49.9%, prevotella 21.1% versus 3.5%, and faecalibacterium 4.8% versus 2.9% (Fig. 1C).

### α- and β-diversities in the gut microbiome of KAHSs and HMPHSs

**FIG. 2.**
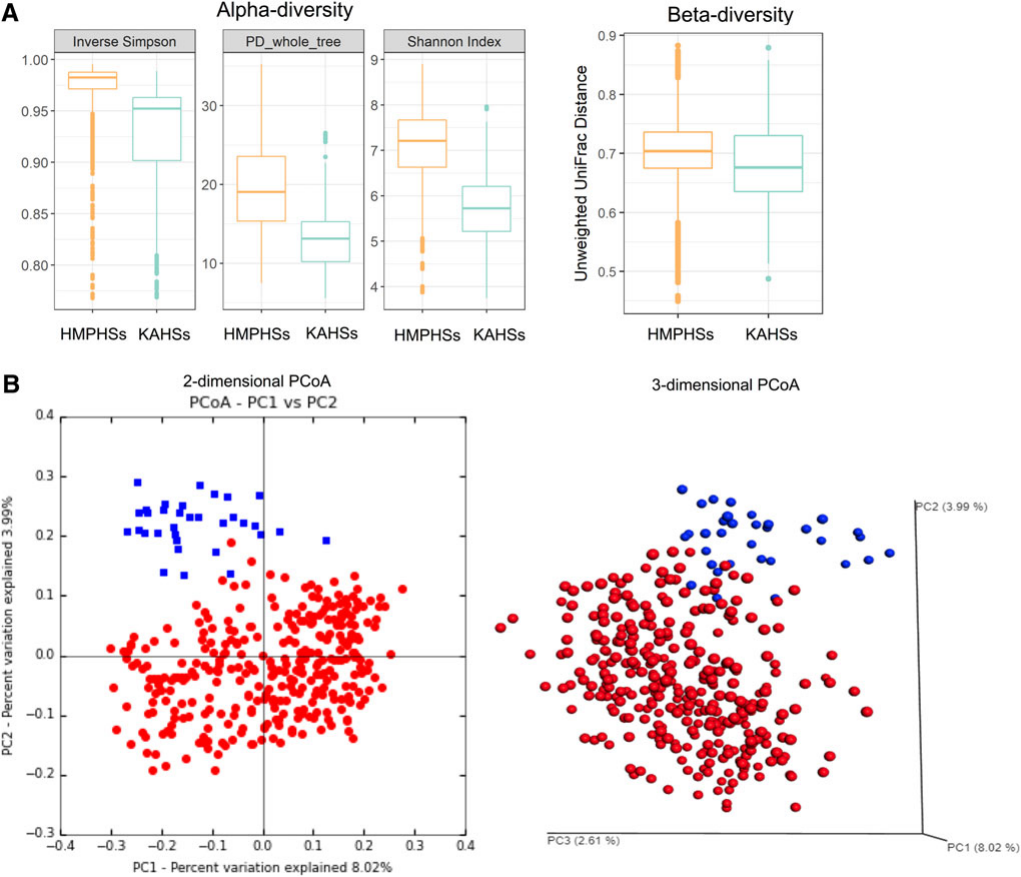
Microbiota diversity estimation of KAHSs and HMPHSs. **(A)** Alpha-diversity estimation showing that the inverse Simpson index for KAHSs was 0.927; for HMPHSs, 0.973. The PD whole_tree index for KAHSs was 13.31; for HMPHSs, it was 19.38; and the Shannon indices were 5.714 for KAHSs and 7.091 for HMPHSs, respectively. Beta-diversity indicated that HMPHSs were higher than KAHSs. **(B)** Two- and three-dimensional PCoA plots for KAHSs and HMPHSs were provided (*blue*: KAHSs; *red*: HMPHSs). Distance metric for the PCoA plots was calculated through unweighted UniFrac distance. PCoA, Principal Coordinate Analysis.

The relative abundance of gut taxa was measured for KAHSs and HMPHSs, in the terms of alpha- and beta-diversities. Alpha-diversities of KAHSs were less than those of HMPHSs (Fig. 2A) in the three measurements [inverse Simpson, phylogenetic diversity (PD) whole trees, and Shannon index], implying higher within-sample organismal diversity in HMPHSs. By using unweighted UniFrac distances (Morgan and Huttenhower, 2012) in QIIME (Caporaso et al., 2010), the beta-diversities of KAHSs (average of 0.685) were significantly (*p*-value 3.51e-26) less than those of HMPHSs (average of 0.707).

Moreover, Principal Coordinate Analysis (PCoA) (Caporaso et al., 2010) of the beta-diversities of KAHSs (Caporaso et al., 2010) showed them to separate from HMPHSs (Fig. 2B). Analysis of similarities tests (in QIIME) between KAHSs and HMPHSs were performed using the unweighted UniFrac distance measurement and the weighted UniFrac distance measurement, yielding *p*-values of 0.0099 and 0.0021, respectively. We performed statistical analyses for the OTU differences between KAHSs and HMPHSs. Under false discovery rate cutoff 0.05, 293 out of 21348 OTUs were statistically significant. Twenty out of the 293 are summarized in Supplementary Table S1. Overall, our results indicate that, even in healthy controls, the gut microbiomes of Korean adolescents and HMP populations were different.

### Functional differences between the gut microbiome of KAHSs and HMPHSs

**FIG. 3.**
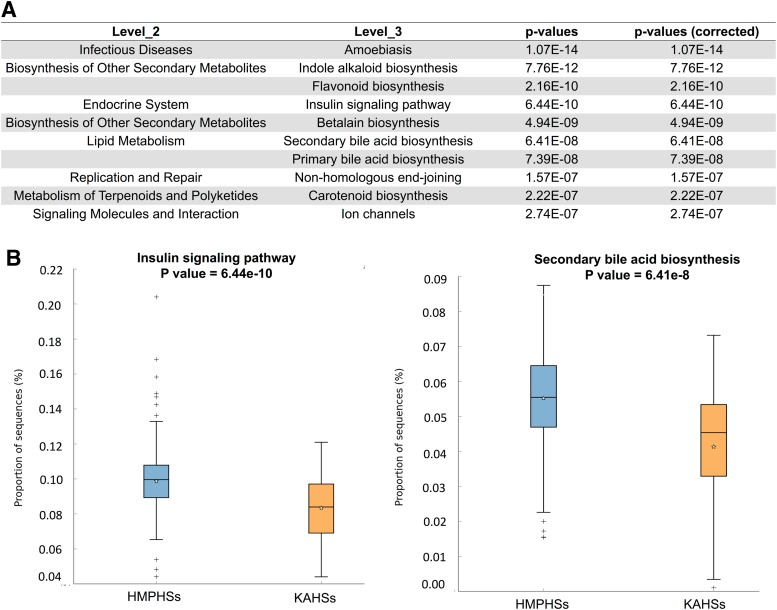
Functional prediction of PICRUSt. **(A)** One hundred and sixty-three significant third-level classification KEGG pathways/terms were reported, based on gastrointestinal microbiota compositions between the two populations (KAHSs and HMPHSs). The 10 significant terms of the 163 are summarized in tabular format. **(B)** Two significant third-level terms (insulin signaling, secondary bile acid synthesis) in **(A)** are drawn as boxplots. KEGG, Kyoto Encyclopedia of Genes and Genomes; PICRUSt, Phylogenetic Investigation of Communities by Reconstruction of Unobserved States.

Using PICRUSt (Langille et al., 2013), functional differences between KAHSs and HMPHSs were predicted. In the first-level classification Kyoto Encyclopedia of Genes and Genomes (KEGG) (Kanehisa and Goto, 2000), pathways of PICRUSt, cellular processes, genetic information processing, and organismal systems were significantly different between KAHSs and HMPHSs (Supplementary Fig. S2) Also, 37 second-level classification KEGG pathways, by PICRUSt, were reported to be significant, and 163 significant third-level classification KEGG pathways, based on the structure of the gastrointestinal microbiota between the two populations. The ten most significant third-level classification KEGG pathways are summarized in Figure 3A. Two of the 10, insulin signaling pathway and secondary bile acid synthesis, are depicted in boxplots in Figure 3B.

Moreover, the abundances of the two pathways of KAHSs were lower than those of HMPHSs. Both bile acids (Ridlon et al., 2014) and insulin signaling (Carvalho et al., 2012) have been recognized as important mediators of health status and well-being of gut microbiota.

### Network comparisons between gut microbiome of KAHSs and HMPHSs

Based on the OTU tables, we constructed microbial correlation networks representing pair-wise associations (equivalently, co-occurrences) among different microbial communities. At a phylum-level network of KAHSs, the largest cluster showed pair-wise associations between bacteroidetes and firmicutes (Supplementary Fig. S3). Also, a HMPHS phylum-level network (Supplementary Fig. S3) showed its largest cluster to link bacteroidetes and firmicutes. Relatedly, recent studies have reported that the relative ratio between bacteroidetes and firmicutes in gut microbiota clinically associates with healthy status and inflammatory diseases (Johnson and Versalovic, 2012; Koenig et al., 2011).

**FIG. 4.**
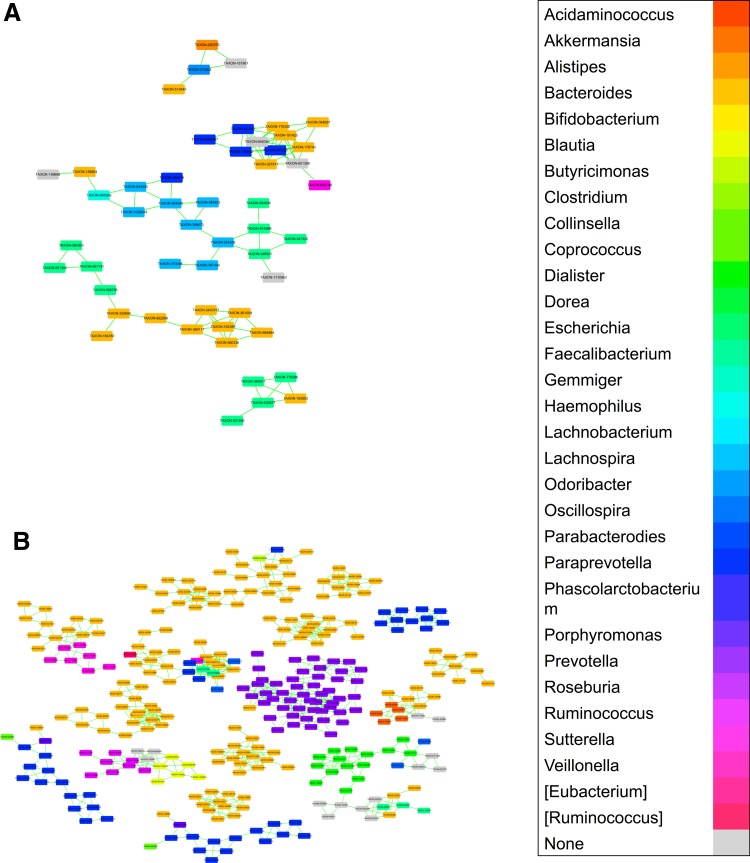
Co-occurrence network among microbial communities of KAHSs and HMPHSs at genus level. **(A)** Co-occurrence networks in KAHSs. **(B)** Co-occurrence networks in HMPHSs. Full networks in genus level for both populations are in Supplementary Figure S4.

Moreover, the abundance ratio of firmicutes/bacteriodetes (0.43) of HMPHSs was higher than that (0.31) of KAHSs, in agreement with another study showing increasing ratios of firmicutes to bacteriodetes during growth into adulthood (Mariat et al., 2009). The second largest clusters of both KAHSs and HMPHSs also indicated that actinobacteria associate with bacteroidetes and firmicutes, respectively (black arrow in Supplementary Fig. S3). Interestingly, in genus-level networks (Fig. 4 and Supplementary Fig. S4), HMPHSs showed more associations among different taxa than KAHSs.

Looking into the different taxa associations (co-occurrence) in Supplementary Figure S4, commonality and difference of the associations between KAHSs and HMPHSs were revealed (Supplementary Fig. S5). HMPHSs had more different taxa associations than KAHSs, indicating that HMPHS microbial communities likely interacted with each other.

### Gut microbiota comparisons of KAHSs with HKAs and IAHSs

**FIG. 5.**
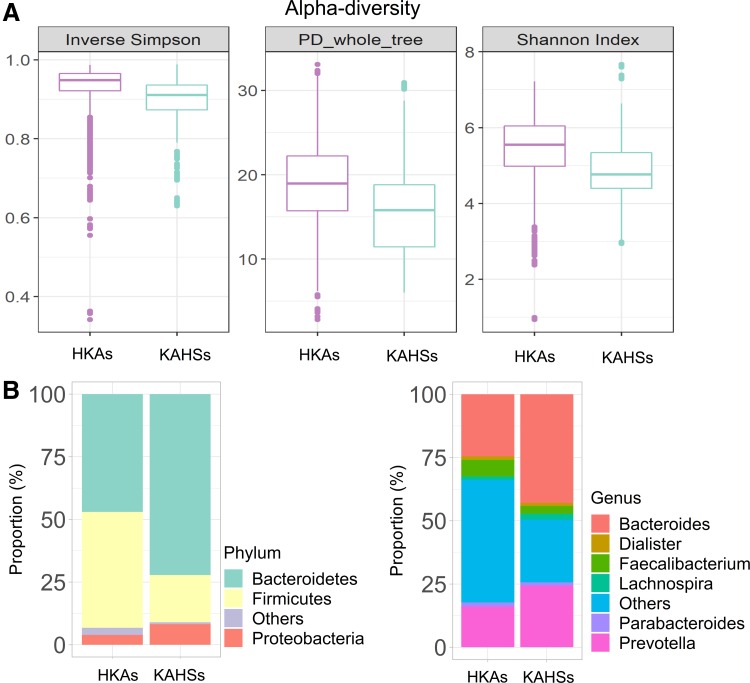
Microbiota comparisons of KAHSs and HKAs. **(A)** Alpha-diversity of KAHSs was lower than that of HKAs. **(B)** Taxonomy composition plots were presented for HKAs and KAHSs. HKAs, healthy Korean adults.

For considering the same population, we compared KAHSs and HKAs (Yun et al., 2017) (Fig. 5A and Supplementary Fig. S6A). Even though two- and three-dimensional PCoA plots indicate that KAHSs and HKAs were not separate (Supplementary Fig. S6A), microbiota diversity was not reached to mature (Fig. 5A).

We further inspected microbiota proportional changes between HKAs and healthy Korean adolescents (KAHSs). At phylum level, from the adolescents (KAHSs) to the adults (HKAs), bacteroidetes were decreased from 72% to 47%, and firmicutes increased from 19% to 46% (Fig. 5B). At genus level, from the adolescents (KAHSs) to the adults (HKAs), bacteroides were changed 43–25%; prevotella 24–16%; faecalibacterium 3–7%; and lachnospira 2–1% (Fig. 5B). These changes from adolescents to adults may demonstrate value for future clinical contexts, not to mention developmental biology.

In addition, to consider an age-matching factor, IAHSs (Del Chierico et al., 2018) were compared with KAHSs. Despite similar ages, the two adolescent cohorts were separate in two- and three-dimensional PCoA plots (Supplementary Fig. S6B). We performed microbiota composition comparison of KAHSs and IAHSs, and found microbiota differences (Supplementary Fig. S7). At phylum level, KAHSs and IAHSs were 71% and 29% for bacteroidetes, respectively; and 20% and 67% for firmicutes, respectively (Supplementary Fig. S7). At genus level, bacteroides were KAHSs 46% and IAHSs 15%; prevotella KAHSs 22% and IAHSs 0.5%; faecalibacterium KAHSs 5% and IAHSs 12%; and lachnospira KAHSs 2% and IAHSs 0.2% (Supplementary Fig. S7).

## Discussion

In this study, we compared gut microbiota between KAHSs and HMPHSs, in terms of microbiota composition (Fig. 1), diversities (Fig. 2), functions/pathways (Fig. 3), and co-occurrence networks (Fig. 4).

Higher ratios of prevotella to bacteroidetes are preferably observed in agrarian diets (i.e., those high in fiber and agricultural products) (Simpson and Campbell, 2015). Of interest, we found the prevotella to bacteroide ratio (0.471) of KAHSs to be higher than that of HMPHSs (0.070) in our study. Such a striking ratio difference likely results from high-fiber ingredients in the Korean diet (Kim et al., 2016).

Recent studies report that the abundance ratio between bacteroidetes and firmicutes, in gut microbiota, clinically associates with both healthy well-being and inflammatory disease (Koliada et al., 2017; Lopez et al., 2016; Verdam et al., 2013). As mentioned earlier, the ratio of firmicutes/bacteroidetes in Korean adolescents (KAHSs) was lower than that in HMP adults (HMPHSs). This may likely indicate that KAHSs have not fully reached mature microbiota composition.

In our PICRUSt analysis (Fig. 3B), we found different abundances of secondary bile acid synthesis between adolescent and adult populations. In this synthetic pathway, secondary bile acids are generated from primary fecal acids (Wong et al., 2006). Recently, this conversion process (i.e., primary to secondary bile acids in fecal samples) was associated with firmicutes and bacteroidetes (Kakiyama et al., 2013). Thus, the significant abundance differences of firmicutes and bacteroidetes between KAHSs and HMPHSs may affect activation of the secondary bile acid synthesis pathway. Also, considering the role of the gut microbiome as a regulator of bile acids (Ridlon et al., 2014; Sayin et al., 2013; Wahlstrom et al., 2016), cholesterol and bile acid pools may be different between KAHSs and HMPHSs.

Insulin signaling is also related to microbiota diversity (Sonnenburg and Backhed, 2016), since diverse SCFAs (e.g., butyrate, acetate), mediated by gut microbiota, associate with increases in insulin sensitivity and AMPK activity (Jiao et al., 2018; Jones, 2016). In that regard, we found that the functional abundance of insulin signaling (Fig. 3B) was lower in KAHSs, than in HMPHSs, again suggesting that microbiota diversity (i.e., alpha- and beta-diversity) of KAHSs are likely still progressing toward the maturity of adult-like microbiota diversity found in HMPHSs. This finding was supported by another comparison of KAHs and HKAs, in that alpha-diversity of Korean adolescents was also lower than that of HKAs (Fig. 5).

Another recent report (Hollister et al., 2015) indicated that microbiota development likely continues over the first 3 years of life, during which gut microbiota are believed to mature, and it is presumed that continuous development of microbiota may make rich associations among different taxa. Consequently, associations among different taxa in KAHSs may not reach the extent of diversities of HMPHSs. In fact, our visual inspection of the two genus-level networks (Supplementary Fig. S4) of KAHSs and HMPHSs showed that adult-like microbiota (HMPHSs) had more associations, among different taxa, than KAHSs.

Since we also targeted the 16S ribosomal RNA (rRNA) regions of KAHSs and HMPHSs, within V1–V3 and V1–V3/V3–V5, respectively (Hu et al., 2015; Human Microbiome Project Consortium, 2012), we concede that different target regions may affect the results, thus warranting careful interpretation of these findings. Also, our study should be carefully interpreted, since microbiota comparison of adolescents and adults from different populations would have unaccounted demographic and other confounding factors.

## Conclusions

In this study, we report the microbiota composition, diversities, functions/pathways, and co-occurrence networks that characterize gut microbiota in Korean healthy adolescents (KAHSs), compared with the large healthy adult cohort, HMPHSs. These differences represent unique characteristics of Korean healthy adolescent gut microbiota. In particular, we noted that KAHSs had a higher ratio of prevotella to bacteroidetes than HMPHSs, with lower microbiota diversity and 163 significantly different KEGG pathways (third level of PICRUSt). Co-occurrences among different taxa happened in HMPHSs rather than in KAHSs. While these differences await further computational and laboratory analysis for robust validation, such approaches will provide insight into the differences between maturing and adult microbial physiology and pathology.

In all, further population studies of microbiome variation across geographies and developmental stages are warranted, and should usefully inform future diagnostics and therapeutics innovation targeting the microbiome.

## Supplementary Material

Supplemental data

## Abbreviations Used

CODA: Clinical & Omics Data Archive
HKAs: healthy Korean adults
HMP: Human Microbiome Project
HMPHSs: Human Microbiome Project healthy samples
IAHSs: Italian adolescent healthy samples
KAHSs: Korean adolescent healthy samples
KEGG: Kyoto Encyclopedia of Genes and Genomes
OTU: operational taxonomic unit
PCoA: principal coordinate analysis
PICRUSt: Phylogenetic Investigation of Communities by Reconstruction of Unobserved States
QIIME: Quantitative Insights Into Microbial Ecology
SCFAs: short-chain fatty acids
