# Supplementary material for: Comparison of Microbiota Variation in Korean Healthy Adolescents with Adults Suggests Notable Maturity Differences
Source: OMICS. 2018 Dec 19;22(12):770–8. doi: 10.1089/omi.2018.0146 (PMC6338580; doi:10.1089/omi.2018.0146)

Comparison of microbiota variation in Korean healthy adolescents with adults suggests notable maturity differences.

Supplementary Information

Supplementary Table 1
Supplementary Figures 1 through 7

Supplementary Table 1. Top 20 for statistically significant different OTUs between KAHSs and HMPHSs .

OTU ID	P-value	FDR_P	KAHSs	HMPHSs	Taxonomy
210155	0	0	71.12121	0.704969	g__Bacteroides
303791	0	0	1.212121	59.96273	g__Bacteroides
356687	0	0	2.393939	194.677	g__Bacteroides
169775	0	0	0.181818	70.47826	g__Bacteroides
4479835	0	0	0	57.68944	g__Bacteroides
260579	0	0	1.606061	63.74845	g__Alistipes
179412	0	0	0.242424	75.6677	g__Bacteroides
4484395	0	0	0	54.01863	g__Bacteroides
183603	0	0	114.7576	6.692547	g__Bacteroides
307571	0	0	81.18182	0.695652	g__Prevotella
130228	0	0	0.666667	56.04348	g__Bacteroides
2097828	0	0	59.66667	0.068323	g__Bacteroides
210939	0	0	111.3333	12.9441	g__Bacteroides
4374912	0	0	0.272727	78.53727	g__Parabacteroides
844958	0	0	7.69697	229.354	g__Bacteroides
292640	0	0	112.7273	11.68323	g__Prevotella
1906483	0	0	0.030303	56.61491	g__Bacteroides
586379	0	0	1.272727	92.70186	g__Bacteroides
360058	0	0	116.1515	0.419255	g__Bacteroides
1935192	0	0	0	52.77019	g__Bacteroides

Supplementary Figure 1. An overview of data analysis in our study.

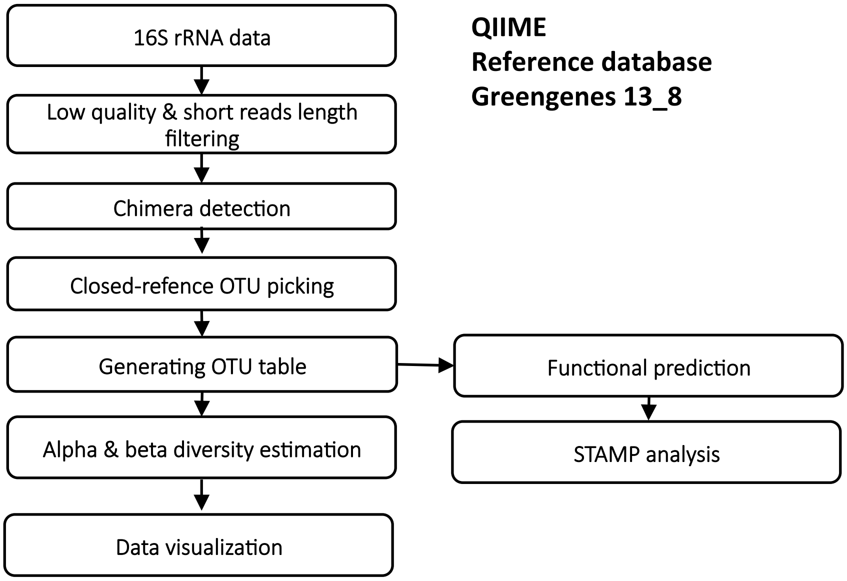

Supplementary Figure 2. PICRUSt functional results of cellular processes, genetic information processing, and organismal system between HMPHSs and KAHSs.

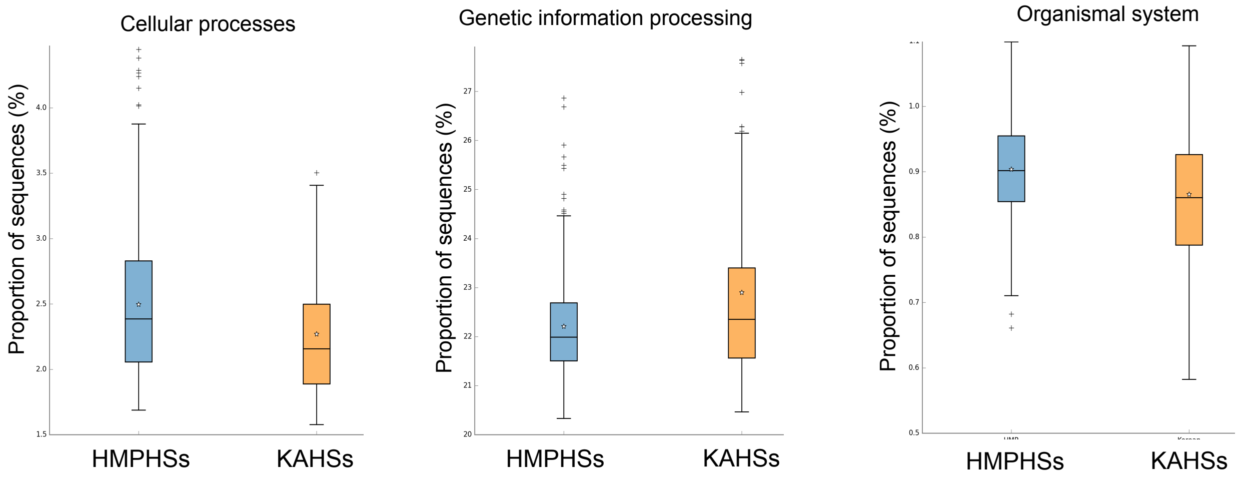

Feature	Eta-squared	P-value	Corrected p-value
Cellular processes	0.025	1.56e-3	1.56e-3
Genetic information processing	0.043	3.38e-5	3.38e-5
Organismal system	0.032	3.80e-4	3.80e-4

Supplementary Figure 3. Phylum-level association (co-occurrence) networks of KAHSs and HMPHSs.

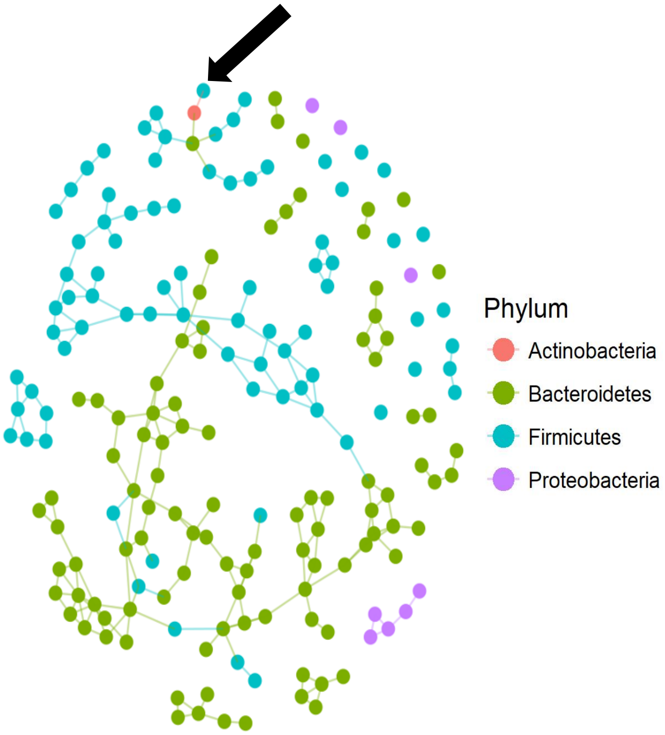

KAHSs – Phylum level

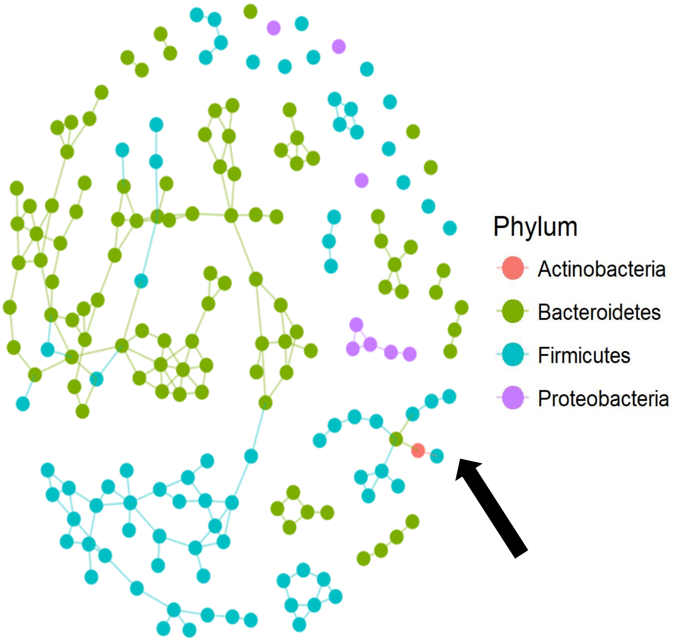

HMPHSs – Phylum level

Supplementary Figure 4. Full genus-level association networks of KPHSs and HMPHSs. In the networks, edges indicates associations among taxa in terms of co-occurrences of neighboring taxa. Figure 4 was a subset of this figure.

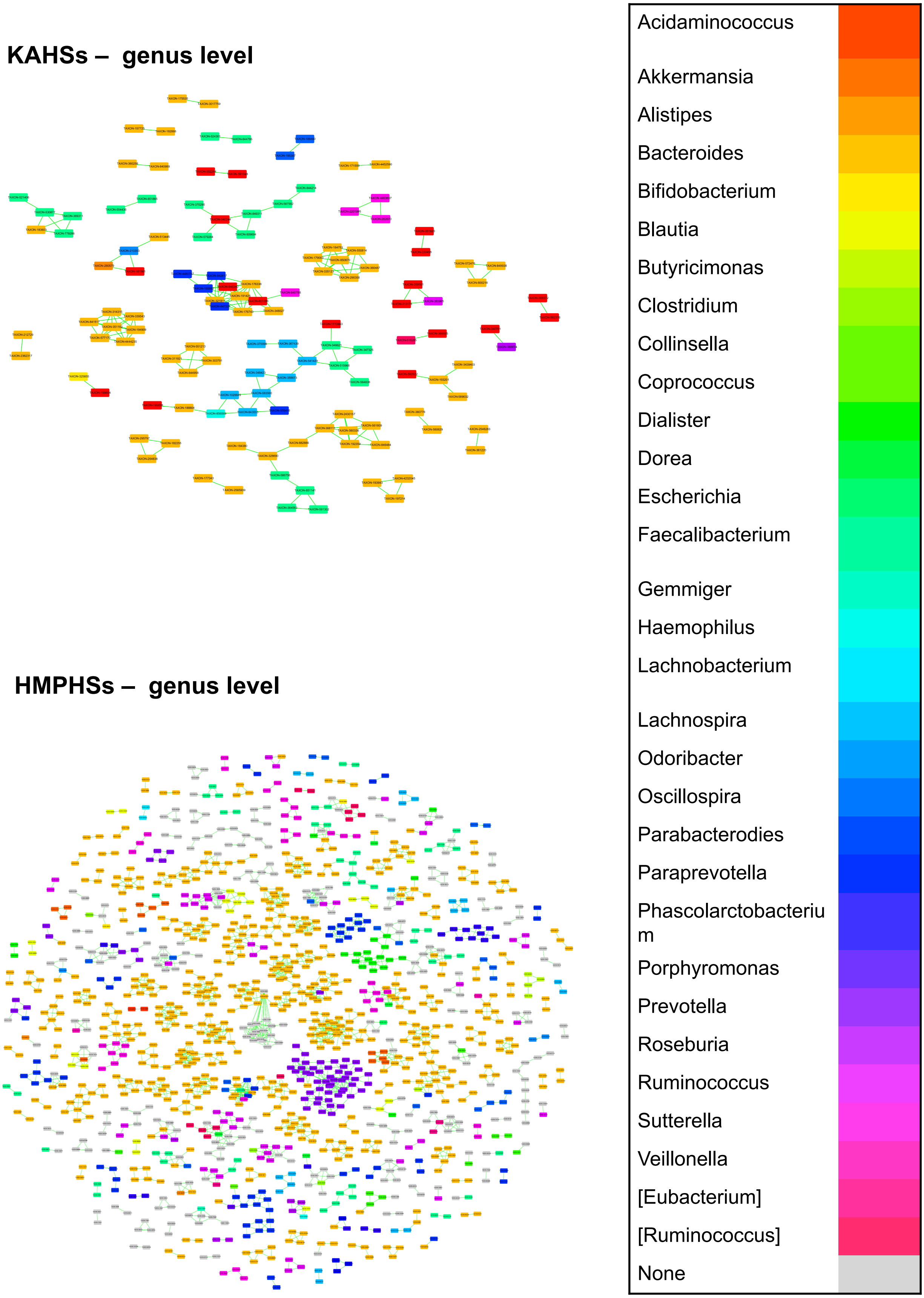

Supplementary Figure 5. Associations among different taxa in genus level networks of KAHSs and HMPHSs. For example, the number of intersection is three which indicate three association among different taxa: bacteroides-faecalibacterium, parabacteroides-sutterella, and bacteroides-parabacteroides.

Bacteroides-Faecalibacterium
Parabacteroides-Sutterella
Bacteroides-Parabacteroides

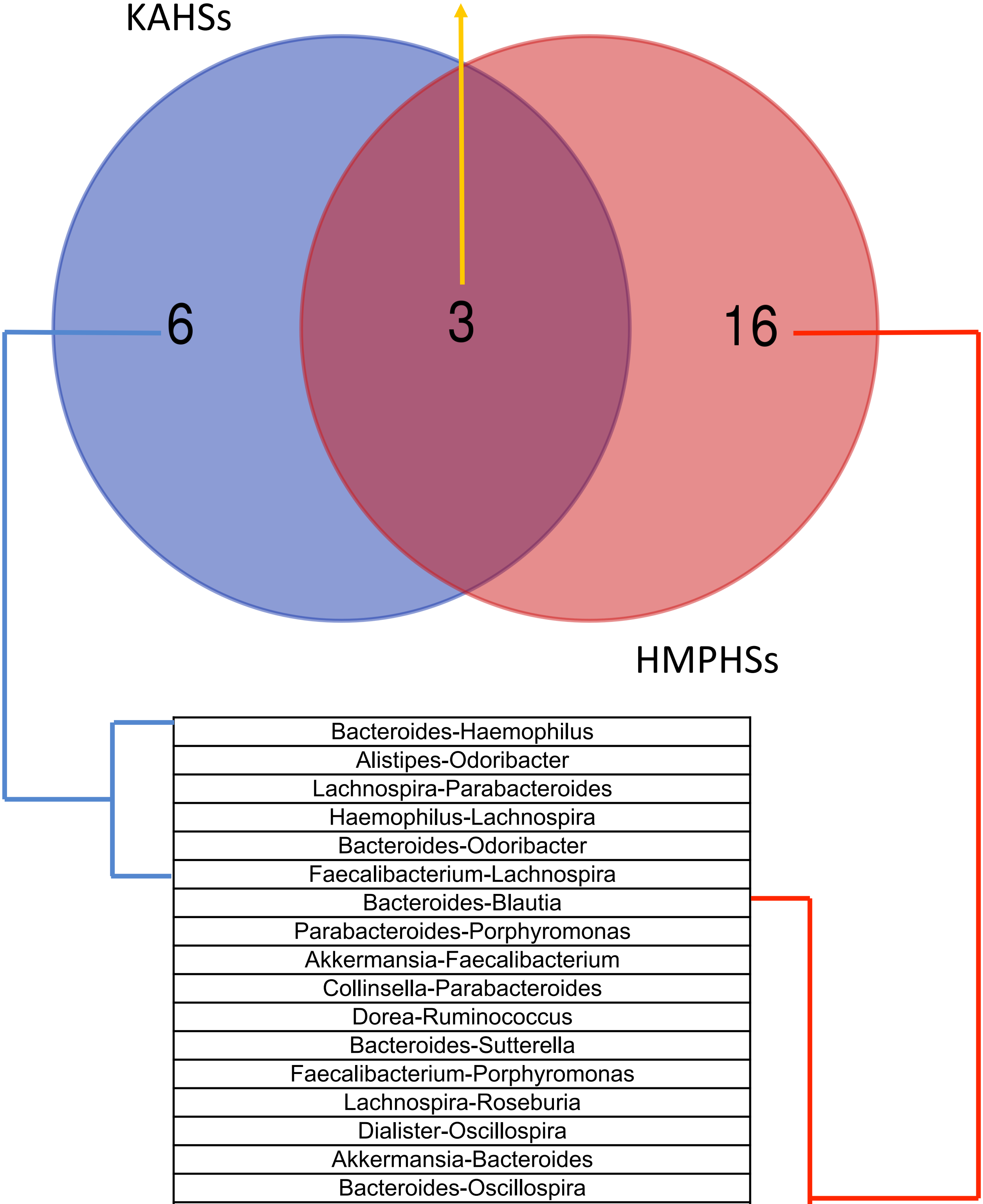

Bacteroides-Haemophilus
Alistipes-Odoribacter
Lachnospira-Parabacteroides
Haemophilus-Lachnospira
Bacteroides-Odoribacter
Faecalibacterium-Lachnospira
Bacteroides-Blautia
Parabacteroides-Porphyromonas
Akkermansia-Faecalibacterium
Collinsella-Parabacteroides
Dorea-Ruminococcus
Bacteroides-Sutterella
Faecalibacterium-Porphyromonas
Lachnospira-Roseburia
Dialister-Oscillospira
Akkermansia-Bacteroides
Bacteroides-Oscillospira
Bacteroides-Porphyromonas
Faecalibacterium-Parabacteroides
Bacteroides-Lachnospira
Blautia-Ruminococcus
Bacteroides-Prevotella

Supplementary Figure 6. PCoA plots for comparing (A) KAHSs with HKAs, and (B) KAHSs and IAHSs. KAHSs and HKAs are closely clustered more than KAHSs and IAHSs.

A

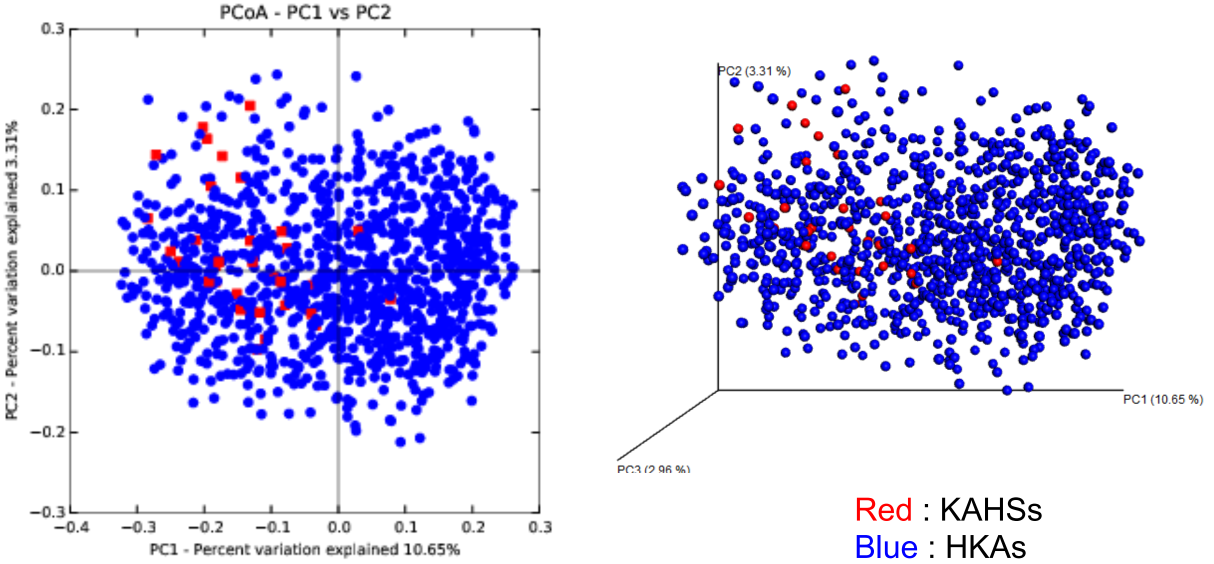

B

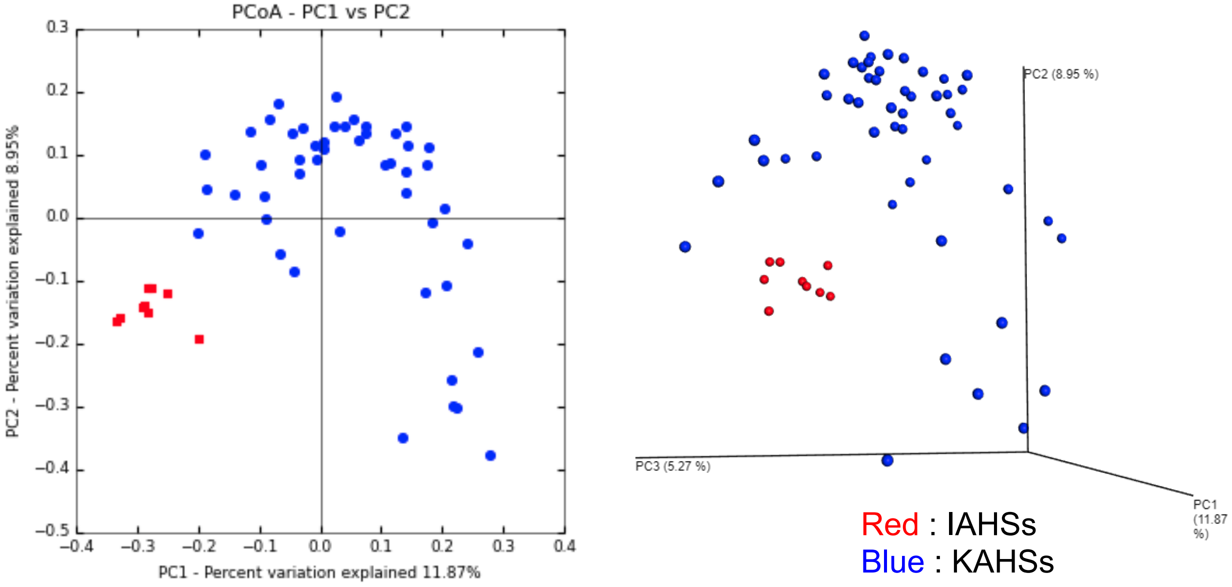

Supplementary Figure 7. Taxonomy composition plots for IAHSs and KHASs.

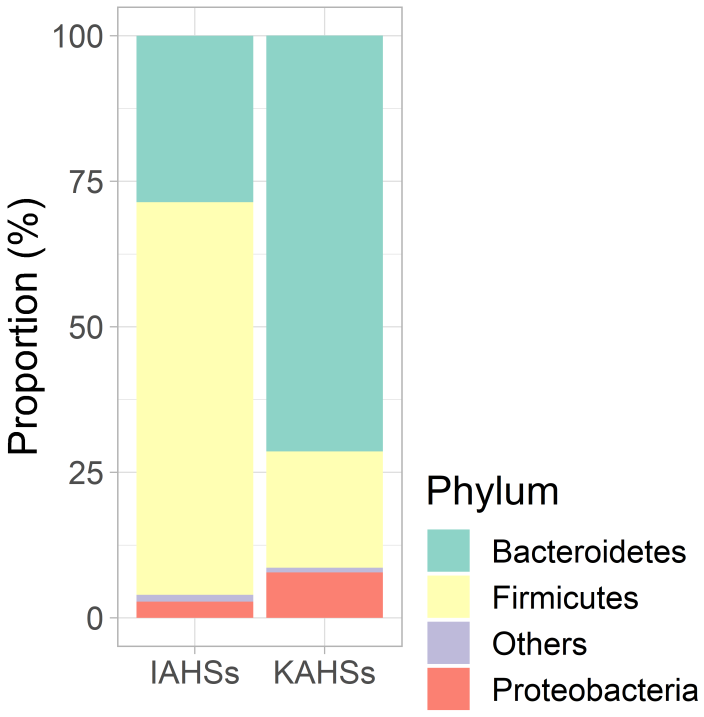

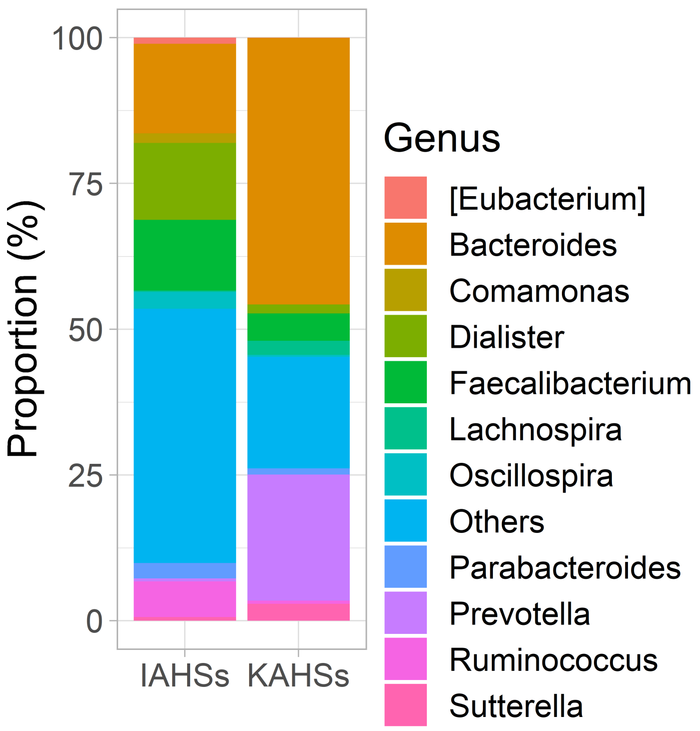

Supplement: Supplemental data [file Supp_Data.pdf]
